# Vibrations in CDFW

**DOI:** 10.3390/e22060704

**Published:** 2020-06-24

**Authors:** Daniel Soares de Alcantara, Pedro Paulo Balestrassi, José Henrique Freitas Gomes, Carlos Alberto Carvalho Castro

**Affiliations:** 1 Institute of industrial Engineering, Federal University of Itajubá (UNIFEI), Itajubá 37500-903, Brazil; 2 Federal Center of Technological Education of Minas Gerais (CEFET-MG), Varginha 37022-560, Brazil

**Keywords:** friction, welding, mean square root, crest factor, peak, kurtosis, empirical mode decomposition

## Abstract

Continuous drive friction welding is a solid-state welding process that has been experimentally proven to be a fast and reliable method. This is a complex process; deformations in the viscosity of a material alter the friction between the surfaces of the pieces. All these dynamics cause changes in the vibration signals; the interpretation of these signals can reveal important information. The vibration signals generated during the friction and forging stages are measured on the stationary part of the structure to determine the influence of the manipulated variables on the time domain statistical characteristics (root mean square, peak value, crest factor, and kurtosis). In the frequency domain, empirical mode decomposition is used to characterize frequencies. It was observed that it is possible to identify the effects of the manipulated variables on the calculated statistical characteristics. The results also indicate that the effect of manipulated variables is stronger on low-frequency signals.

## 1. Introduction

Currently, different techniques allow metallic and non-metallic materials to be joined by welding. Among the many existing processes, friction welding (FW) is a technology that has been drawing interest due to its various advantages compared to conventional welding techniques [1,2,3,4,5]. Friction welding is a solid-state technique where heat generation is achieved by the friction between pieces or between pieces and a non-consumable auxiliary tool. For friction generation, an axial force is applied along with a cycle of rotary or linear (oscillatory) motion to at least one of the pieces or to the auxiliary tool. Due to the movement used in the generation of the heat flow, the FW is divided into rotary friction welding (RFW), linear friction welding (LFW), and, with auxiliary rotary tools, stir friction welding (FSW). We consider these to be the fundamental processes and the origin of the other FW variants. RFW has two very similar variants, inertia friction welding (IFW) and continuous drive friction welding (CDFW), whose differences lie in their process control and equipment. These variants have two stages, friction and forging [6,7]. Some researchers also include intermediate stages [8,9,10]. CDFW’s main manipulated variables are axial pressure, time, and rotational speed. Similarly, IFW’s variables are axial pressure, time, rotational speed, and also the mass and volume of the kinetic energy generator. In CDFW, rotational speed is provided at all stages by an electric motor. This feature allows for more precise control. In the IFW, there is a flywheel that generates the inertial rotation speed; the electric motor is decoupled from the workpiece, disallowing precise control. In the CDFW process, researchers usually aim to develop or adapt the equipment to their work requirements. Hence, four types of machines are typically applied: the adapted lathe, the computer numerical controlled (CNC) lathe, lathes specially manufactured for these processes, and those built by researchers. We found in our survey that 59% of machines are adapted equipment. For IFW, this adaptation involves more than simply dedicating the equipment to the task. Considering these factors, among the rotary processes, CDFW is the simplest; however, this apparent simplicity is misleading. A combination of manipulated variables generates complex physical, metallurgical, and thermal phenomena.

There are also fluctuations in the roughness, coefficient of friction, and time-varying undulations. Due to these effects, as well as the fact that the machine-piece set has specific deformations, a complex and variable vibration system is developed during the process. Depending on the parameters used in the process, different vibration intensities are generated. At this particular point, vibration signals are suitable for detecting process-specific conditions [11]. Several studies have been conducted to explain and improve the quality of joints, but no systematic analyses of vibrations in the CDFW process were found in the published literature. In summary, many articles describe the benefits of vibrations in welded joints rather than during the process. The study in [12] researched vibrations in rotary friction welding. This work is unique in trying to study vibrations, but the focus of the research was on the origin of the oscillations that accumulate inside a piece. Therefore, understanding the vibrations on the CDFW process is essential and could help reduce possible mechanical damage to the equipment, increase its efficiency, reduce costs, and provide improvements in joint properties. The main objective of this study is to characterize the CDFW process vibrations in the structures of the stationary parts to determine their time-domain statistical characteristics (root mean square, peak value, peak value, crest factor, and kurtosis) and to characterize the influence of each manipulated variable. To identify the influences of the manipulated variables, this study used an experimental design and analysis of variance (ANOVA). In the frequency domain, the signals were analyzed by the empirical mode decomposition method (EMD) in order to identify the frequencies generated during the process.

## 2. CDFW Process

**Figure 1:**
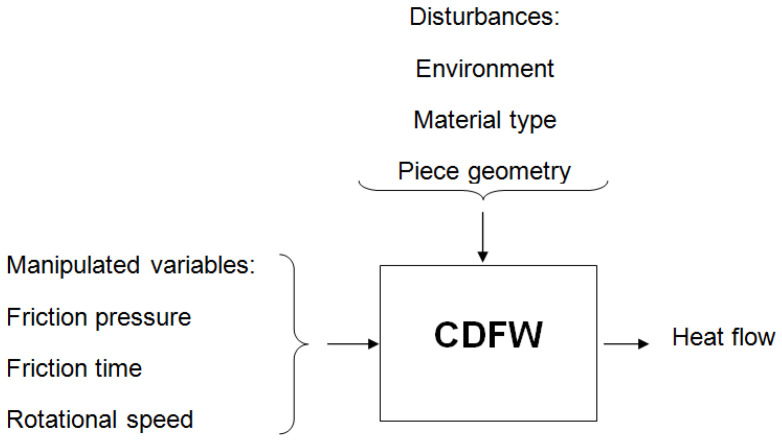
Continuous drive friction welding (CDFW) control flowchart.

The CDFW process uses the following control flowchart, represented in Figure 1:

Manipulated variables are the process variables that influence the response of interest and can be changed by the process control system. These disturbances can also influence the response of interest but cannot be altered by the process control system. In turn, the parameters are the quantification of the manipulated variables. Parameters are the factors that can be adjusted to control a process; each parameter is important to the process, and one’s knowledge can improve the process’s quality and properties. In all FW processes, the topic of interest is heat generation, which alters various materials and junction properties [13]. Due to the fast nature of this process, the process is commonly defined in two stages: the friction stage and the de-forging stage [6,14].

**Figure 2:**
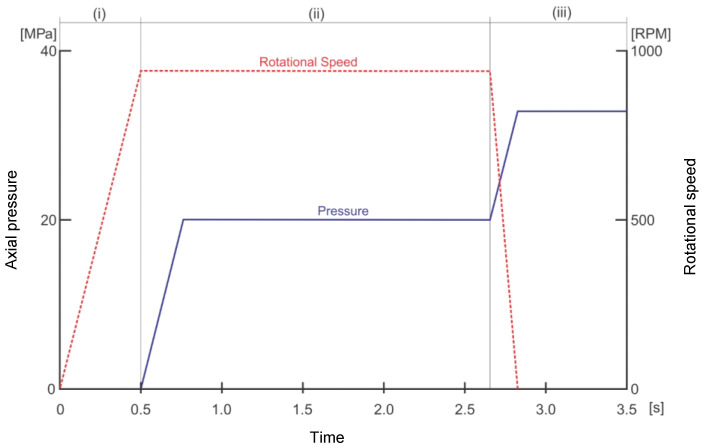
Stages of the CDFW process [10,17].

Figure 2 presents a definition of the three stages and the main process parameters involved in each stage [15,16]. For ease of analysis, the friction stage has been divided into three parts: (i) the preheating stage, (ii) the friction stage, and (iii) the forging stage.

**Figure 3:**
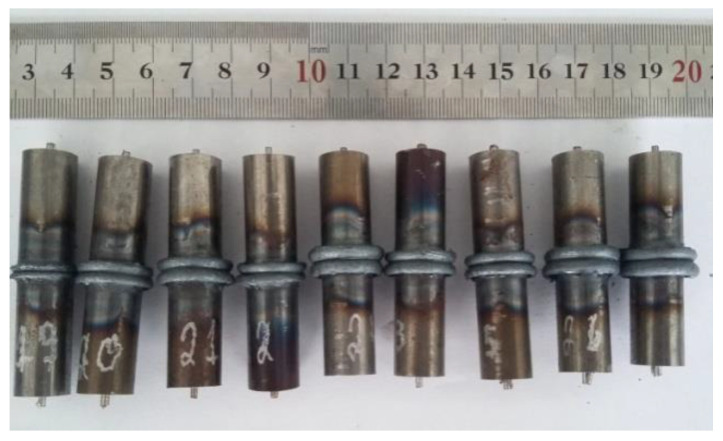
CDFW junction.

In Figure 2, the “preheating stage”, when the two parts are still in contact with no pressure applied, the piece’s rotation speed increases linearly to a suitable maximum value. In the initial period, the contact between the pieces is practically in a solid-state. Next, in the “friction stage” the rotational speed is kept constant (i.e., the axial friction pressure) and increases up to a certain value and then remains constant. In this stage, thermal energy and plastic deformation are generated due to friction. Because of the gradual softening, the softened material radially flows out to form the flash and starts the burn-off [10]. In the next stage, after a certain amount of friction time, the rotational speed starts to decrease until it reaches a value of zero; the friction pressure then retains its maximum value. The relative movement between the pieces ceases, and the friction pressure is replaced by forging pressure until the reduction of the length (burn-off) is complete. In this stage, heat is produced by applying forging pressure on the plastic material. During this stage, chemical interdiffusion occurs, and the plastic material continues to be expelled [18,19]. Figure 3 shows an image of a CDFW welded ASTM A35 rod. The joint reveals flash formation on both sides of the joint, which is a typical feature of friction welding.

Therefore, to produce a high-quality welding joint, it is essential to understand the influencing factors and determine the best parameter settings. These parameters together with the properties of the base material, such as the diameter, surface finish, deformation resistance, fusion point, base metal ductility, and hardness, influence the material’s flow on the interfacial layer [20,21].

In the radial direction, due to the decrease in velocity from the periphery to the center of the workpiece, the temperature distribution is irregular. In the axial direction, the shorter the distance from the surface, the higher the rate of temperature increase and the higher the peak temperature [22]. Thus, the system conditions may change during this process. The variations in each of these stages produce periodic and random vibration waves, which provide information to monitor changes in the equipment and process.

## 3. Vibration Signals in CDFW

Vibrations in industrial processes have been a major concern for researchers, as each process and each piece of equipment generates its own vibrational identity [23]. In CDFW, it is evident that as the surfaces come into contact, the friction causes wear. It is also evident that this wear plasticizes the surface material and that the plastic state of the material alters the contact area between the pieces. All these dynamics cause changes in the vibration signals. It is worth remembering that the CDFW equipment is a rotating machine that is strong enough to withstand the friction pressure and torque caused during the process and also contributes to the generation of vibration signals. In line with the above, during CDFW, vibrations may be as caused by various sources. The mechanisms of possible sources of vibration are:

(1) Plastic deformation during the piece heating process;
(2) Friction contact between pieces;
(3) Friction contact between the workpiece and the mandrel;
(4) Collisions between the generated flash and the environment;
(5) Rotational movement of the piece.

According to the definitions of the types of mechanical vibrations presented in [24], we propose that the initial frictional contact between the pieces, and the plastic deformation that occurs during the process, can impact and cause free vibration in the system. Friction contact between the workpiece and the mandrel, collisions between the generated flash and the environment, and the rotational motion of the workpiece can cause forced vibration. However, this effect needs to be better studied, and little is known about the vibrations in CDFW.

## 4. Vibration Signal Analysis

Analyzing the signs of vibrations has been successfully used in various industrial processes [25,26]. Various methods were used by researchers to analyze vibration signals [27,28,29]. Due to the complexity and dynamics of the signals, studies have been conducted to establish the criteria for processing the vibration signals of the CDFW process. The greatest difficulty lies in separating the signals generated by the equipment from the signals generated by the CDFW. Proper filters should be used to eliminate most of the noise embedded in the signal. In the time domain, amplitude and frequency are the characteristic parameters of the signals. For analysis, the signal’s descriptive parameters and statistics provide much information [30]. This is a widely used type of analysis and has the possibility to directly process the vibration signals and perform simple mathematical and statistical calculations [31]. Time-domain analysis is based directly on the temporal waveform itself. The characteristics usually extracted from these signals are mean square root (RMS), peak value (PV), kurtosis (KT), and crest factor (CF) [32,33]. These statistical characteristics reveal the signal’s energy intensity, which may reveal a change of friction in CDFW. The RMS value indicates the energy level of the vibrations in the manufacturing processes; this value is used to detect unbalanced elements. The peak represents the highest value of the process vibration’s RMS signal. The CF value is used to predict some imbalances in rotating components. KT is a widely used tool for the detection of hidden impulses in a signal [34]. Time-domain resource formulas are listed in Table 1.

In the frequency domain, the density and power characteristics have been studied. There are several methods to extract information from the vibration signals of industrial processes [35,36,37,38]. Among these methods, empirical mode decomposition (EMD) is applied to signals with non-stationary characteristics [39,40]. EMD acts as a filter and decomposes the signal into several ranges called the intrinsic mode function (IMF) component. Decomposed signals have frequency characteristics of the original signal called IMF. The first IMFs are high-frequency signals, and with the advance of decomposition, low-frequency IMFs are generated [1]. EMD is an efficient method for decomposing vibration signals. A detailed explanation of EMD can be found in [41,42].

## 5. Materials and Methods

**Figure 4:**
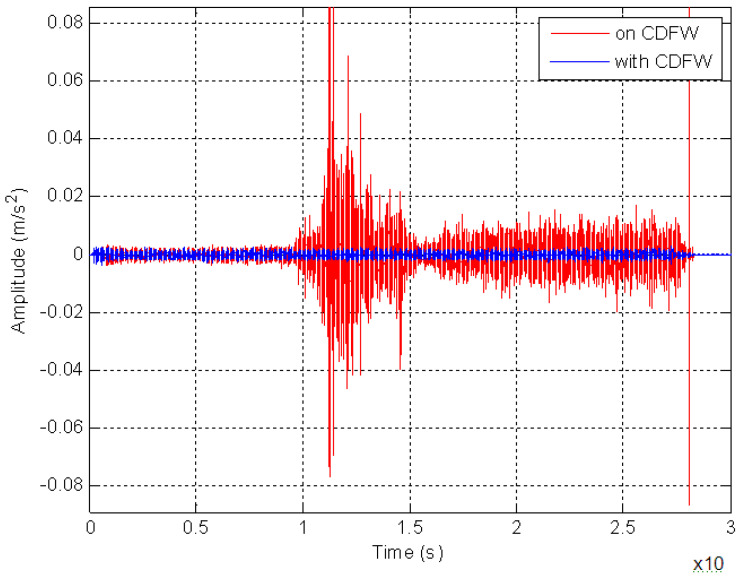
Overlapping time signal of the vibration with and without the CDFW friction load.

To try to characterize the process vibrations, CDFW equipment was developed using a platform with a conventional lathe. Modifications in the electronic control to automate the system and provide a programmable platform for speed variation were performed. A new speed control design based on a frequency inverter was proposed. This design allowed for variable speeds up to 3000 rpm. To provide the power required to apply axial force (friction/forging pressure) to workpieces, during the CDFW process, modifications were made to the drive structure and hardware (mechanical drive). A torque generating mechanism with a gearbox and a DC servo motor were installed, allowing for the application of a downward force up to 1 kN. In addition, a microcontroller was installed to provide precise control of the friction time and forging time. This circuit acts as an external trigger for the measuring equipment to ensure that it always begins measuring at a set time. The workpiece material used throughout the experiments was ASTM A36 steel, which is commercially available in solid bars with a diameter of 12.7 mm × 60 mm. The choice of ASTM A36 steel was made due to its good weldability, low mechanical strength, and low machinability. The sample surfaces prior to welding were machined using a conventional lathe to eliminate the effect of surface roughness. The obtained pieces were used to produce the CDFW joints. An accelerometer used to acquire the vibration signals during the process was installed perpendicularly in the X–Y direction (a resonant type sensor with a frequency response between 10 and 10,000 Hz (ICP PCB 357B11, no. 5500B)). The device was connected to a data acquisition card operational amplifier (DAQ, NI cDAQ-6062) using a BNC-2120 connector accessory. This device-generated many resources to gain the most information about vibrations during the process. The signals emitted by the accelerometers were acquired for the observation time according to each process stage and sampled at 5000 Hz. The collected data were stored directly on a PC hard disk using LabView software. After data acquisition, the vibration signal was conditioned using an antialiasing filter. In addition, an IIR filter with an order of 5 and a cutoff frequency of 3 kHz was selected for all vibration signals to filter the vibration signal to eliminate noise from the CDFW machine. To select the usable frequency band, the time domain signal energy behavior of the CDFW equipment without the friction load was taken into account. When the CDFW process begins operation, the equipment’s natural frequency is excited, resulting in an increase in power. This pulse is superimposed—that is, its amplitude is modulated—on a carrier signal originating from the rotary machine. The behavior of the time domain signals is shown in Figure 4.

**Figure 5:**
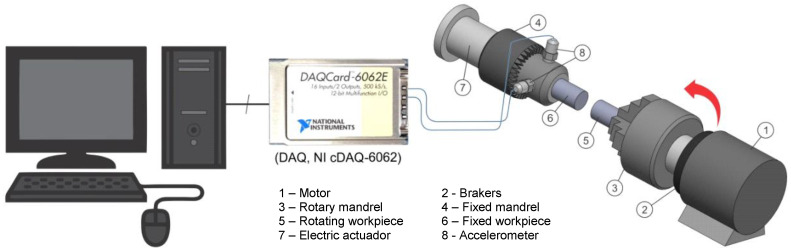
Experimental setup.

Figure 4 clearly shows that the CDFW process significantly affected the overall vibration of the equipment signal, so the amplitude of vibration increased substantially. The dynamic characteristics of the process produced dynamic vibration characteristics. To verify these characteristics, it is necessary to examine the effects of the input parameters that may influence the vibration level. A complete factorial design was selected to prepare the experiments. Preliminary tests were performed to select the parameter levels in order to obtain a good weld without defects. Figure 5 shows the experimental configuration used to obtain the signals to be analyzed. As shown in this figure, the accelerometers have been fixed to the workpiece without rotation.

Table 2 shows the experimental design matrix and presents the factors and levels used in the experiment. In this approach, four factors with two levels each were considered (high (1) and low (−1)), totaling 16 experiments. For each test, the CDFW process was performed, and the vibration signals were collected and recorded.

**Figure 6:**
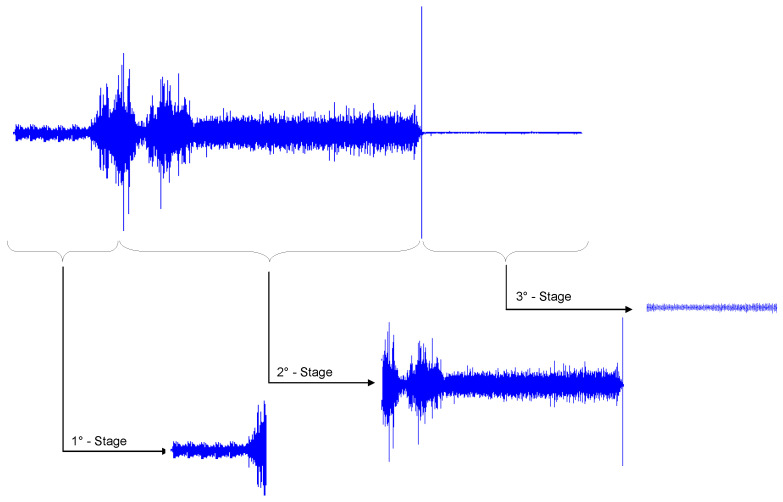
Full vibration signal and signals corresponding to the CDFW stages.

In order to reduce the dimensionality of the data and determine the stages with the highest vibration, the total signals were divided according to the CDFW process stages (described in Section 2). In this experiment, an initial preheating stage was added to the process to identify changes in the vibration signals without applying axial force. The decomposition results are presented in Figure 6, which shows the decomposed temporal signals. The windows were based on a variation of the times of each stage established by the factorial design.

**Figure 7:**
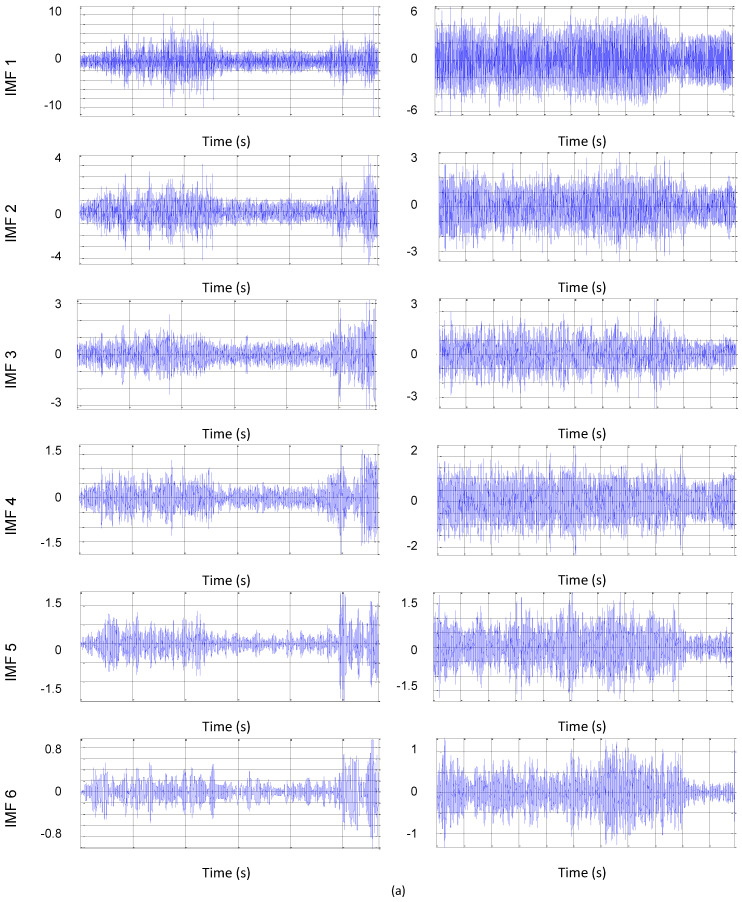

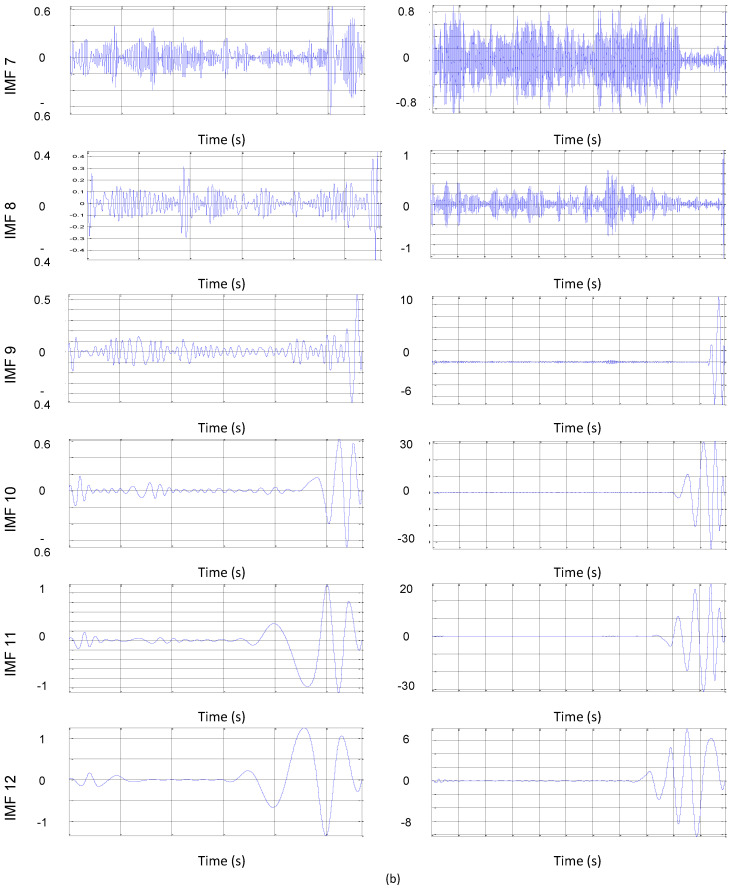
Intrinsic mode function (IMF) plots, (**a**) stage 1, and (**b**) stage 2.

As shown in Figure 6, the CDFW process can be divided into three stages. The first stage occurs after preheating the workpieces; the rotation is applied, and gradually the axial force is increased. The initial warm-up time was 5 s in all experiments. As shown, the initial translational and rotational impact vibrations between the two workpieces are of low amplitude. The second stage begins with the application of the axial force at its maximum value, and contact between the workpieces is made. The third and last stage is related to the completion of the welding process; only the forging force is applied in this stage. Deceleration at the end of the process has been shown to cause additional amplitude in the vibration signal. As shown in Figure 6, the waveforms have various frequency components that belong to the various parameters that are pieces of the CDFW process. These vibrations originate from the translational and rotational impact between pieces [12]. The decomposition of the signals in these stages is appropriate when treating each process stage as a separate signal. For each stage, the experiments were performed according to the planning matrix. Then, preprocessing was performed, during which the parameter estimation routines were performed. The detection of radial vibration through accelerometers directly measures acceleration, and, according to [12], both axial and tangential vibrations are transferred to a radial movement due to the conservation of volume. This allows one to capture both oscillations with a sensor in the radial direction. The experimental amplitude values (RMS) in the radial direction (X) were used for the statistical processing and monitoring of each of the process stages. Using software, the analysis focused on determining which stages had the highest energy and which parameters had the greatest influence on the statistical characteristics of the vibration signals. The signals were processed and analyzed in the time domains to extract potential resources. A total of four features were extracted from the time domain signal, including the root mean square (RMS), peak (PK), crest factor (CF), and kurtosis (KT). Then, using the statistical software, the effects of the manipulated variables on the calculated statistical values were verified with a variance test (ANOVA). To determine the effects of the manipulated variables on the frequency domain vibration signal, an EMD algorithm was used to decompose the temporal signal into frequency bands. For each experimental run, 12 IMFs were generated, totaling 192 IMFs for each stage. We opted for this amount to ensure the efficiency of decomposition [43]. However, the data processing time was high. Figure 7 shows the decomposition signals based on an experimental run.

To optimize the processing time, only the RMS value was calculated to identify the effects of the manipulated variables in the IMF. Then, to verify the efficiency of the EMD method, the instantaneous frequency of the IMF was calculated. To generate the effects graph, the 4 IMFs with the highest and lowest frequencies were used. Changes in IMF signals are easily identified based on amplitude and frequency.

## 6. Results and Discussion

**Figure 8:**
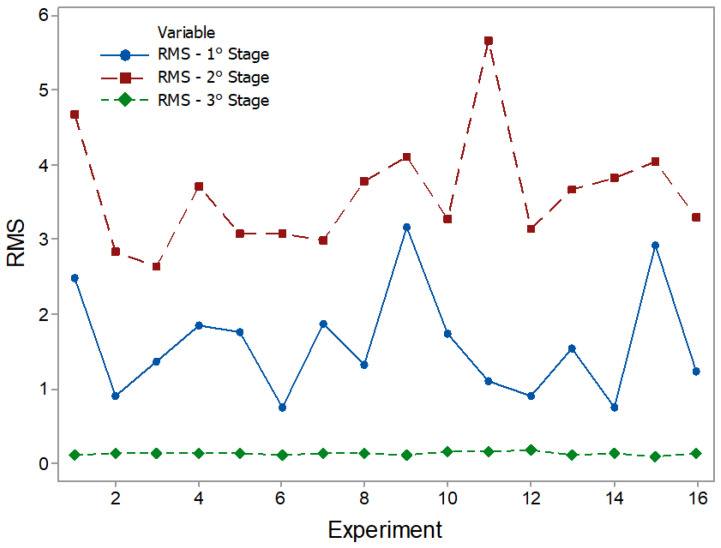
RMS plots.

**Figure 9:**
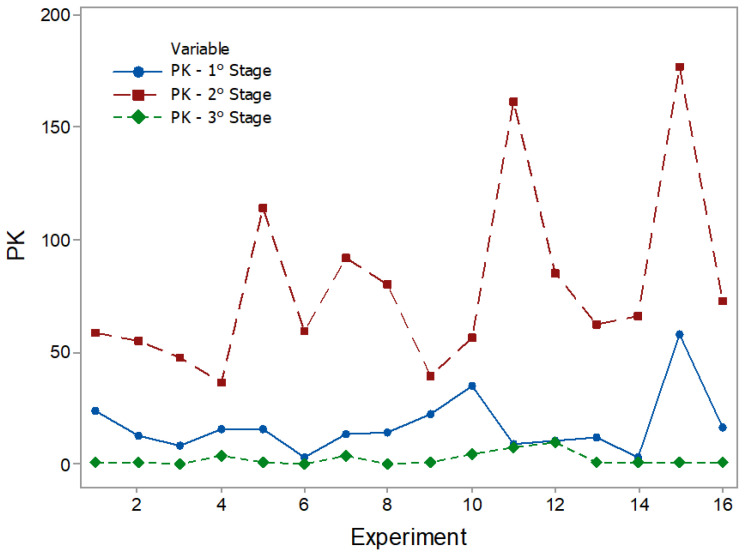
PK plots.

**Figure 10:**
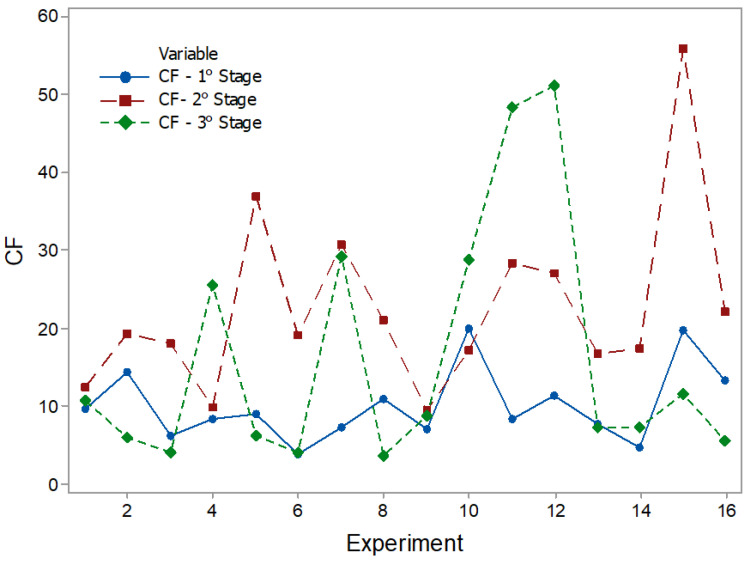
CF plots.

**Figure 11:**
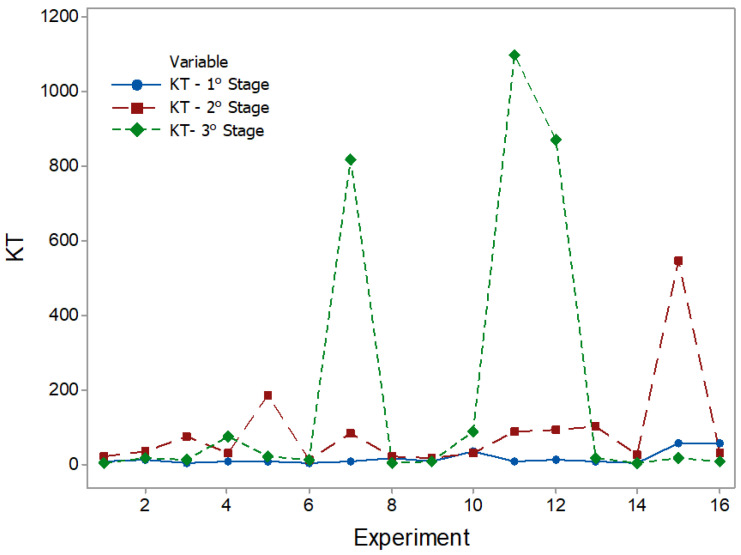
KT plots.

The results of the statistical characteristics for RMS, PK, CF, and KT are shown in Figure 8, Figure 9, Figure 10 and Figure 11 for all experimental conditions at each stage of the process.

The results for RMS and PK show the highest and most sudden variations during the second stage, where the applications of axial force and rotation speed are constant. On average, the second stage signals were approximately twice as high as the first stage signals. This result is in line with the research in [44]. During the first stage of the process, the contact area between the surfaces of the workpiece was only about 10% of the nominal area of the friction interface and was increased with the increasing axial force during the second stage. This, in turn, resulted in greater friction between the surfaces, causing greater vibrations. In the third stage, there was practically no sign of vibrations. However, fluctuations can be observed in PK values. These fluctuations were likely caused by significant mechanical deformation due to forging [19]. This phenomenon can also be observed in CF and KT. In the present work, the statistical characteristics of stage 3 will not be analyzed.

Table 3, Table 4, Table 5, Table 6, Table 7, Table 8, Table 9 and Table 10 show the ANOVA results for RMS, PK, CF and KT, respectively. In Table 3, for stage 1 RMS, the results show that the greatest variation in vibration comes from the RPM (39.11%), followed by the interaction of PF * TF (27.27%) and RPM * TF (7.24%). The values of F also confirm this trend. By analyzing the value of P in the same way, the RPM and PF * TF are observed to be statistically significant factors. This is expected since, at the beginning of the RPM process, they are the only parameters that reach their maximum interaction values. This observation is in agreement with the results in [19], which indicates the strong influence of the combined factors in the process. However, the individual terms, PF and TF, did not present a significant influence. Stage 2 (Table 4) shows the influence of RPM * TF interactions (46.47%). This combination of factors makes the biggest contribution to vibration generation. The importance of RPM (19.85%) is once again confirmed, followed by TF (7.47%). The values of F also confirm this tendency. By analyzing the value of P in the same way, RPM and RPM * PF are shown to significantly generate vibrations. Next (Table 5), we analyzed the PK. In stage 1, we can observe that the behavior of PK is different from that of the RMS. The largest variation is observed for the interaction PF * TF (33.46%), then for the RPM * TF (12.01%), and individually for RPM (6.36%). However, only PF * TF is significant (P < 0.05). In stage 2 (Table 6), PF (15.02%), RPM (14.79%), and TF (8.48%) were the most influential individual parameters, followed by the combination RPM * PF (7.04%). We observed that the combinations PF * TF (36.03%) and RPM * TF (22.55%) are the most significant factors affecting the CF value in stage 1 (Table 7). In addition, we observed that these factors are statistically significant (P < 0.05). As CF is the ratio between RMS and PK, this result confirms the strong influence of these parameters in stage 1. However, RPM, PF, and TF, had minimal effect. Likewise, in stage 2 (Table 8), the percentage contributions of the parameters TF, RPM * TF, PF, and RPM were, respectively, calculated as 18.98%, 13.32%, 13.00%, and 9.72%. However, none were significant. By using ANOVA on KT (Table 9, stage 1), we observed that the most influential factor is the interaction PF * TF (31.69%), followed by PF (9.99%). However, a significant effect (P > 0.05) was observed only for PF * TF. In stage 2 (Table 10), RPM (17.37%) and RPM * TF (16.21%) were the most influential factors, but there was no significant effect (P > 0.05).

**Figure 12:**
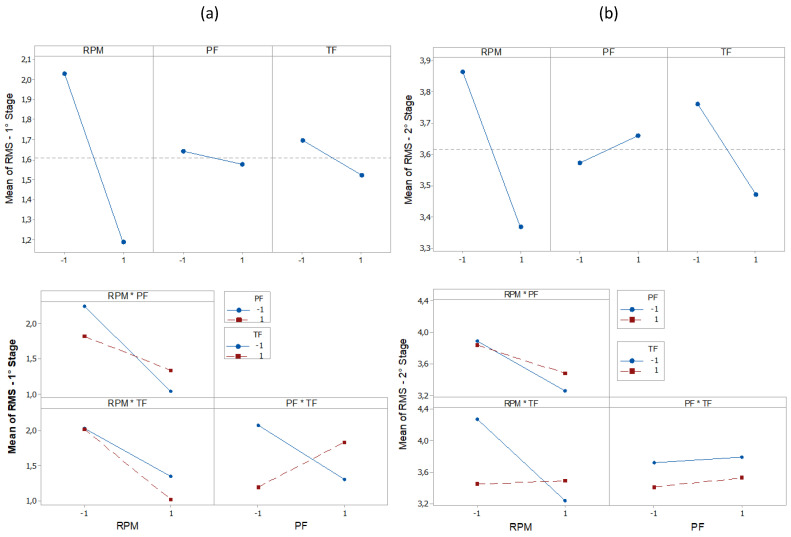
Effect and Interaction plots of the vibration RMS, (**a**) stage 1 (**b**) stage 2.

**Figure 13:**
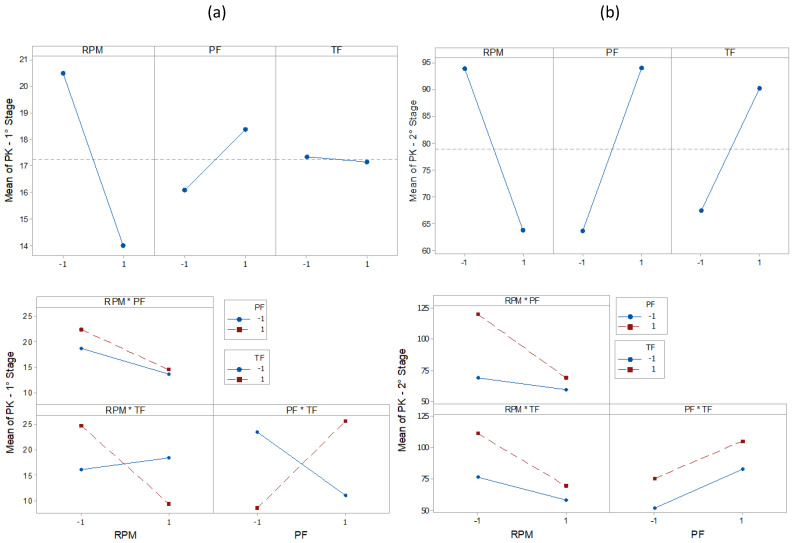
Effect and Interaction plots of the vibration PK, (**a**) stage 1 (**b**) stage 2.

**Figure 14:**
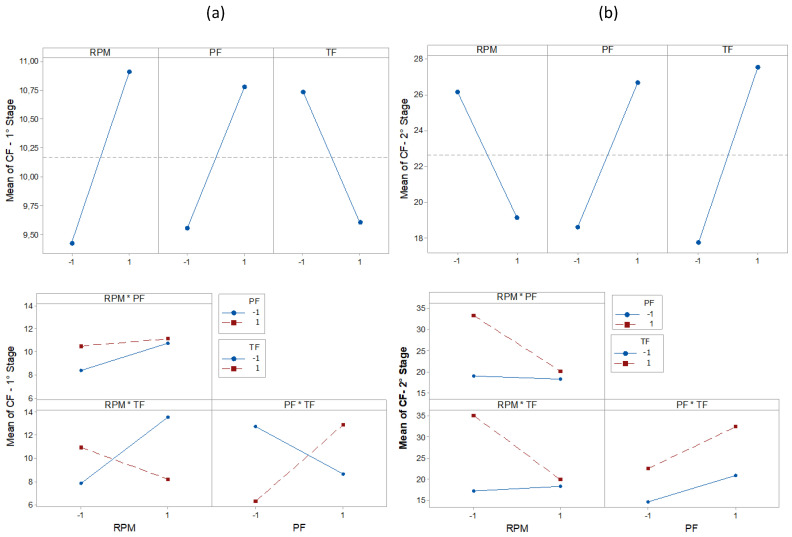
Effect and Interaction plots of the vibration CF, (**a**) stage 1 (**b**) stage 2.

**Figure 15:**
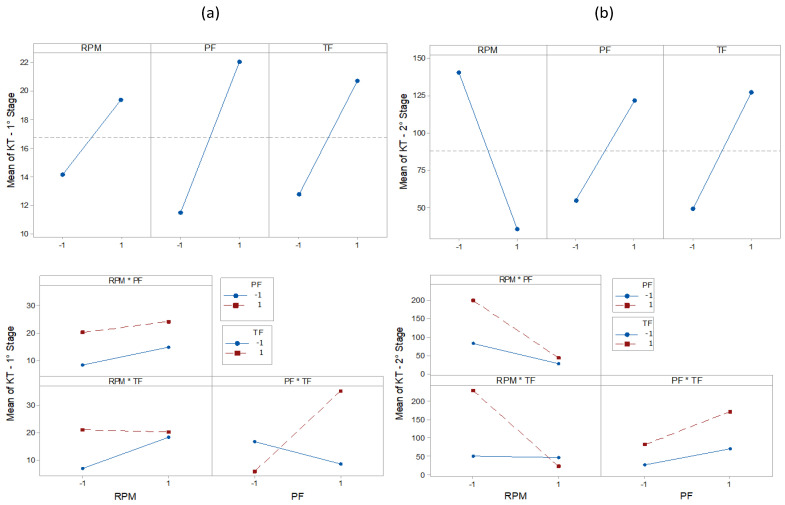
Effect and Interaction plots of the vibration KT, (**a**) stage 1 (**b**) stage 2.

The main effect plot for RMS, PK, CF, and KT is shown in Figure 12, Figure 13, Figure 14 and Figure 15. The influence of RPM on stage 1 is confirmed again, as shown in Figure 12a. The RMS value decreases with increasing speed. Increasing the RPM causes an increase in temperature such that the material is plasticized, and friction is replaced by a polishing action [15]. This also causes changes in the coefficient of friction [10]. Therefore, as the RPM increases, the friction is reduced, causing low vibration intensity. The interaction graph shows the strong influence of RPM, whose effect does not depend on other parameters. In turn, changes in PF and TF levels had no effect. In stage 2 (Figure 12b), the RPM effect maintains the same trend as that in stage 1. With constant speed, the heat generation mechanism is activated, which leads to the production of a larger volume of viscous material. The vibration then tends to decrease. It is noteworthy that an increase in signal strength relative to stage 1 is again confirmed, likely caused by the increased contact area between surfaces due to the application of PF. In addition, it was found that RMS is proportional to PF and inversely proportional to TF. In Figure 13a, the effects of the parameters on PK are shown. In stage 1, the lowest values were observed with an increase of RPM and with low values of PF. In stage 2 (Figure 13b), the RPM maintained the same trend. However, an increase in PF and TF increased the vibration peaks. This phenomenon correlates with the research by [45]. Long-time and high-friction pressure increase the flash output, which creates a larger contact area, thereby producing mechanical deformation and generating vibration peaks. A higher PF also results in more compressed plastic material, causing peaks in the vibration [46]. The CF value in stage 1 (Figure 14a) is low for lower RPM and PF values, with a high value for TF. In stage 2 (Figure 14b), the CF value is low for high RPM and also low for low PF and TF values. These results indicate that the CF is sensitive to variations in the RPM and TF, as confirmed by the interaction graph. In stage 1 (Figure 15a), the KT value decreases by increasing all parameters. In stage 2 (Figure 15b), this trend is different only for the RPM, which produces low KT at a high RPM.

Based on the analysis of the IMF signals in Figure 9, the noise of these signals is concentrated in the high frequencies of both stages. Table 11 shows that, for the RMS of the IMF from each stage of the first experiment, the responses to the other experiments were very similar, and most of the energy is in the second stage. According to this analysis, the energy of the second stage IMFs is higher compared to the energy of the first stage. Theoretically, this energy differential is expected due to the results presented by the time domain RMS analysis. Analyzing the amplitudes (Figure 9) shows that, in the preheating stage, the IMF is higher compared to the IMF 1 and IMF 2 of the forging stage. From IMF 3, the amplitudes begin to decrease in the first stage, and the inverse effect occurs in the forging stage. In summary, in the early stage, high frequencies present greater amplitudes compared to low frequencies. In the final stage, low frequencies present greater amplitudes compared to the high ones. This tendency can be explained by the physical phenomena of each stage, as already discussed in this study. In the forging stage, due to the high heat flux at the periphery of the workpiece, the friction action is replaced by a sliding action, generating low pulses at a high frequency. Near the center of the piece, this sliding action is smaller, generating high pulses at a low frequency.

**Figure 16:**
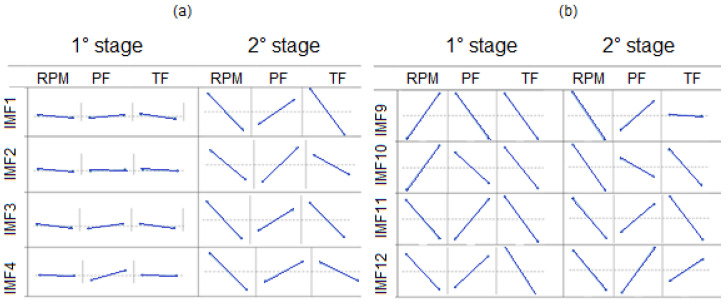
Effect plots of the RMS, (**a**) 1° stage (**b**) 2° stage.

As a result, it is likely that the vibration signals increase in amplitude from the periphery to the center of the piece and that the plasticized material determines these characteristics. It can be seen in IMF that the first stage is characterized by the presence of multiple frequency signals. The effects of the manipulated variables for each IMF were also analyzed (Figure 16 shows the RMS effects chart). As a result, the vibration signals likely increase in amplitude from the periphery to the center of the piece and that the plasticized material determines these characteristics. It can be seen in the IMFs that the first stage is characterized by the presence of multiple frequency signals.

Notably, there is a low influence of the manipulated variables in the high-frequency signals of the first stage. In the second stage, the effects showed the same tendency. Among the low-frequency signals, this trend remained in the first stage of TF and in the second stage of RPM.

## 7. Conclusions

It is possible to extract information from the vibration signals of the CDFW process using a simple methodology. In an attempt to verify the influence of the manipulated variables on the vibrations generated by CDFW, the significant influence of the rotational speed was verified, as expected. A high RPM value produces a low vibration amplitude. In turn, high PF values produce high peaks in the vibration amplitude. A long friction time produces a lower vibration amplitude but favors the generation of peaks. The RMS values in the friction stage presented higher vibrational energy than those in the preheating stage. The calculated RMS is a good indicator of the physical and thermomechanical changes in the different CDFW stages. PK has been shown to be sensitive to variable manipulated pressure. CF is sensitive to changes in rotational speed. KT did not reveal much information but was useful in detecting the presence of vibration peaks at all stages of the process. For the low-frequency signals, the effects of the manipulated variables are stronger. It is likely that the vibration signals increase in amplitude from the periphery to the center of the piece and that the plasticized material determines these characteristics. The efficiency of this proposal needs to be verified for other materials and parameters. Its application will be useful in the design of industrial machines and robots using the CDFW process.

## Figures and Tables

**Table 1:** Formulas mean square root (RMS), peak (PK), crest factor (CF), and kurtosis (KT).

Feature Description	Formula	Feature Description	Formula
Root mean square	$\sqrt{\frac{1\;}{N}}\sum_{i=1}^{N}X_{i}^{2}$	Crest factor	$\frac{\mathrm{Peak}\;\mathrm{Value}}{\mathrm{Root}\;\mathrm{mean}\;\mathrm{square}}$
Peak value	$max(X_{i\;})$	Kurtosis	$\frac{1}{N}\sum_{i=1}^{N}{\left(\frac{X_{i}-\bar{X}}{\tau^{4}}\right)}^{4}$

**Table 2:** Experimental parameters of the CDFW process.

Symbology	Factors	Levels	
		−1	1
N	Rotational speed	1250	1600
PF	Friction pressure	30	54
TF	Fiction time	11	19
TS	Forging time	11	19

**Table 3:** Results of ANOVA for RMS, stage 1.

Source	DF	Adj SS	Adj MS	F-Value	P-Value	Contribution (%)
RPM	1	2.82391	2.82391	13.57	0.006	39.11
PF	1	0.01700	0.01700	0.08	0.782	0.24
TF	1	0.11878	0.11878	0.57	0.472	1.64
RPM*PF	1	0.52302	0.52302	2.51	0.151	7.24
RPM*TF	1	0.10462	0.10462	0.50	0.498	1.45
PF*TF	1	1.96953	1.96953	9.47	0.015	27.27
Error	6	1.66429	0.20804			23.05
Total	12	7.22115				

**Table 4:** Results of ANOVA for RMS, stage 2.

Source	DF	Adj SS	Adj MS	F-Value	P-Value	Contribution (%)
RPM	1	0.114784	0.114784	6.27	0.037	19.85
PF	1	0.002241	0.002241	0.12	0.735	0.39
TF	1	0.043182	0.043182	2.36	0.163	7.47
RPM*PF	1	0.268732	0.268732	14.69	0.005	46.47
RPM*TF	1	0.000094	0.000094	0.01	0.945	0.02
PF*TF	1	0.002923	0.002923	0.16	0.700	0.51
Error	8	0.146365	0.018296			25.31
Total	14	0.578321				

**Table 5:** Results of ANOVA for PK, stage 1.

Source	DF	Adj SS	Adj MS	F-Value	P-Value	Contribution (%)
RPM	1	168.22	168.223	1.08	0.329	6.36
PF	1	20.77	20.767	0.13	0.724	0.79
TF	1	0.15	0.153	0.00	0.976	0.01
RPM*PF	1	8.67	8.666	0.06	0.819	0.33
RPM*TF	1	317.59	317.589	2.04	0.191	12.01
PF*TF	1	884.56	884.561	5.69	0.044	33.46
Error	8	1243.52	155.44			47.04
Total	14	2643.48				

**Table 6:** Results of ANOVA for PK, stage 2.

Source	DF	Adj SS	Adj MS	F-Value	P-Value	Contribution (%)
RPM	1	3624.1	3624.08	2.25	0.172	14.79
PF	1	3680.1	3680.10	2.29	0.169	15.02
TF	1	2077.4	2077.35	1.29	0.289	8.48
RPM*PF	1	1725	1724.96	1.07	0.331	7.04
RPM*TF	1	529.5	529.49	0.33	0.582	2.16
PF*TF	1	0.4	0.41	0.00	0.988	0.00
Error	8	12869.1	1608.64			52.51
Total	14	24505.6				

**Table 7:** Results of ANOVA for CF, stage 1.

Source	DF	Adj SS	Adj MS	F-Value	P-Value	Contribution (%)
RPM	1	8.850	8.850	0.65	0.442	2.790952898
PF	1	5.995	5.995	0.44	0.525	1.890594646
TF	1	5.110	5.110	0.38	0.556	1.611499357
RPM*PF	1	2.922	2.922	0.22	0.655	0.921487499
RPM*TF	1	71.519	71.519	5.28	0.051	22.55436839
PF*TF	1	114.279	114.279	8.43	0.02	36.03924364
Error	8	108.421	13.553			34.19185357
Total	14	317.096				

**Table 8:** Results of ANOVA for CF, stage 2.

Source	DF	Adj SS	Adj MS	F-Value	P-Value	Contribution (%)
RPM	1	195.41	195.41	2.13	0.183	9.723680478
PF	1	261.30	261.296	2.85	0.130	13.00239348
TF	1	381.48	381.481	4.16	0.076	18.98259879
RPM*PF	1	156.37	156.374	1.70	0.228	7.78103432
RPM*TF	1	267.85	267.854	2.92	0.126	13.32832412
PF*TF	1	12.75	12.748	0.14	0.719	0.634445147
Error	8	734.47	91.809			36.54752367
Total	14	2009.63				

**Table 9:** Results of ANOVA for KT, stage 1.

Source	DF	Adj SS	Adj MS	F-Value	P-Value	Contribution (%)
RPM	1	108.70	108.70	0.42	0.535	2.45
PF	1	443.20	443.20	1.71	0.227	9.99
TF	1	252.86	252.86	0.98	0.352	5.70
RPM*PF	1	6.11	6.11	0.02	0.882	0.14
RPM*TF	1	146.62	146.62	0.57	0.473	3.30
PF*TF	1	1406.16	1406.16	5.43	0.048	31.69
Error	8	2072.89	259.11			46.72
Total	14	4436.54				

**Table 10:** Results of ANOVA for KT, stage 2.

Source	DF	Adj SS	Adj MS	F-Value	P-Value	Contribution (%)
RPM	1	44,028	44,028	3.09	0.117	17.37
PF	1	17,858	17,858	1.25	0.296	7.05
TF	1	24,255	24,255	1.70	0.228	9.57
RPM*PF	1	10,030	10,030	0.70	0.426	3.96
RPM*TF	1	41,070	41,070	2.88	0.128	16.21
PF*TF	1	2080	2080	0.15	0.712	0.82
Error	8	114,094	14,262			45.02
Total	14	253,415				

**Table 11:** RMS corresponding to IMF by stage, (**a**) 1° stage, (**b**) 2° stage.

(a)												
Experiment	IMF	IMF	IMF	IMF	IMF	IMF	IMF	IMF	IMF	IMF	IMF	IMF
	1	2	3	4	5	6	7	8	9	10	11	12
1	2.01	0.86	0.55	0.37	0.28	0.20	0.14	0.09	0.09	0.14	0.36	0.44
2	1.65	1.00	0.85	0.48	0.37	0.32	0.91	5.32	14.42	11.13	8.07	19.92
3	1.29	0.92	0.74	0.57	0.70	0.84	0.40	0.26	0.45	1.28	1.16	4.09
4	3.29	1.51	1.02	0.73	0.53	0.49	0.23	0.18	0.33	0.71	1.12	0.84
5	1.95	1.05	0.71	0.57	0.43	0.30	0.22	0.14	0.10	0.09	0.07	0.06
6	4.01	2.02	1.22	0.90	0.69	0.60	0.39	0.24	0.50	0.86	0.74	0.53
7	1.51	0.87	0.55	0.41	0.36	0.28	0.17	0.12	0.25	1.01	0.96	0.36
8	1.79	1.16	0.87	0.98	0.91	0.87	0.37	0.27	0.31	0.72	3.01	2.61
9	2.57	1.50	0.90	0.64	0.43	0.45	0.33	0.43	0.59	1.56	6.14	13.92
10	1.42	0.84	0.46	0.32	0.25	0.20	0.12	0.13	1.48	2.79	1.64	1.47
11	4.23	1.89	1.50	1.07	0.95	0.80	0.48	0.72	0.24	0.35	30.40	47.82
12	0.77	0.50	0.40	0.50	0.51	0.44	0.27	0.61	2.84	4.65	3.09	8.27
13	1.23	0.66	0.48	0.41	0.36	0.64	0.69	1.23	2.77	2.34	2.06	2.92
14	0.61	0.36	0.26	0.21	0.16	0.13	0.27	0.70	1.95	1.74	1.10	1.29
15	2.47	1.59	0.67	0.67	0.58	0.64	0.34	0.27	0.32	0.78	1.11	1.53
16	0.93	0.59	0.40	0.37	0.37	0.30	0.38	0.90	0.71	2.89	5.65	3.65
**(b)**												
**Experiment**	**IMF**	**IMF**	**IMF**	**IMF**	**IMF**	**IMF**	**IMF**	**IMF**	**IMF**	**IMF**	**IMF**	**IMF**
	**1**	**2**	**3**	**4**	**5**	**6**	**7**	**8**	**9**	**10**	**11**	**12**
1	2.34	0.97	0.78	0.66	0.52	0.37	0.30	0.15	1.28	6.53	6.78	2.13
2	3.25	1.77	1.51	1.12	0.87	0.88	0.61	0.37	0.21	0.22	1.11	2.59
3	1.85	1.03	0.86	0.76	0.57	0.48	0.62	0.24	0.58	0.42	0.45	0.77
4	2.49	1.38	0.89	0.69	0.53	0.42	0.30	0.21	0.14	0.28	0.31	0.29
5	2.47	1.25	0.86	0.77	0.72	0.54	0.43	0.30	0.36	1.00	1.88	2.04
6	2.14	1.31	0.90	0.80	0.67	0.69	0.56	0.39	0.29	0.49	2.75	6.64
7	2.08	1.25	1.00	0.84	0.82	0.71	0.53	0.33	0.22	0.24	0.42	1.21
8	2.66	1.65	1.19	1.02	0.83	0.62	0.44	0.29	0.31	0.60	0.79	0.70
9	3.00	1.86	1.39	1.18	0.88	0.76	0.87	0.42	0.27	0.47	0.82	0.93
10	2.54	1.42	1.18	1.00	0.71	0.61	0.43	0.32	0.16	0.62	2.91	2.73
11	3.35	1.83	1.67	1.37	1.13	0.97	0.95	0.60	1.39	3.30	2.19	12.94
12	2.42	1.38	0.93	0.80	0.65	0.52	0.39	0.44	0.49	1.40	1.53	3.70
13	2.53	1.52	1.22	1.11	1.00	0.88	0.69	0.37	0.24	0.19	0.26	0.34
14	3.36	1.94	1.47	1.03	0.81	0.62	0.47	0.32	0.22	0.34	1.43	1.88
15	3.28	2.01	1.47	1.19	1.07	0.92	1.38	1.32	1.91	2.84	1.49	1.95
16	2.47	1.41	1.06	0.98	0.79	0.55	0.41	0.30	0.18	0.24	4.76	5.72
